# Septicemia after chemotherapy for childhood acute lymphoblastic leukemia in China: A multicenter study CCCG‐ALL‐2015

**DOI:** 10.1002/cam4.2889

**Published:** 2020-01-28

**Authors:** Yiping Zhu, Rong Yang, Jiaoyang Cai, Jie Yu, Yanjing Tang, Yumei Chen, Ningling Wang, Hailong He, Xuedong Wu, Frankie W. T. Cheng, Lirong Sun, Yingyi He, Xiuli Ju, Xin Tian, Qun Hu, Runming Jin, Kaili Pan, Yongjun Fang, Xiaowen Zhai, Hui Jiang, Chi‐kong Li

**Affiliations:** ^1^ Key Laboratory of Birth Defects and Related Diseases of Women and Children (Sichuan University) Ministry of Education Department of Pediatric Hematology/Oncology West China Second Hospital of Sichuan University Chengdu China; ^2^ Department of Hematology/Oncology Shanghai Children's Medical Center Shanghai Jiaotong University of School of Medicine Shanghai China; ^3^ Department of Hematology/Oncology Children's Hospital of Chongqing Medical University Chongqing China; ^4^ State Key Laboratory of Experimental Hematology and Division of Pediatric Blood Diseases Center Institute of Hematology and Blood Disease Hospital Chinese Academy of Medical Sciences and Peking Union Medical College Tianjin China; ^5^ Department of Pediatrics Anhui Medical University Second Affiliated Hospital Hefei China; ^6^ Department of Hematology/Oncology Children's Hospital of Soochow University Suzhou China; ^7^ Department of Pediatrics Nanfang Hospital Southern Medical University Guangzhou China; ^8^ Department of Paediatrics Hong Kong Children's Hospital The Chinese University of Hong Kong Hong Kong SAR China; ^9^ Department of Pediatrics Affiliated Hospital of Qingdao University Qingdao China; ^10^ Department of Hematology/Oncology Guangzhou Women and Children Health Care Center Guangzhou China; ^11^ Department of Pediatrics Qilu Hospital of Shandong University Jinan China; ^12^ Department of Hematology/Oncology Kunming Children's Hospital Kunming China; ^13^ Department of Pediatric Hematology Tongji Hospital of Tongji Medical College Huazhong University of Science and Technology Wuhan China; ^14^ Department of Pediatrics Union Hospital of Tongji Medical College Huazhong University of Science and Technology Wuhan China; ^15^ Department of Hematology/Oncology Xi'an Northwest Women and Children Hospital Xian China; ^16^ Department of Hematology/Oncology Nanjing Children's Hospital Affiliated to Nanjing Medical University Nanjing China; ^17^ Department of Hematology Oncology Children's hospital of Fudan University Shanghai China; ^18^ Department of Hematology Oncology Children's Hospital Affiliated to Shanghai Jiaotong University Shanghai China

**Keywords:** acute lymphoblastic leukemia, children, mortality, multicenter study, septicemia

## Abstract

**Background:**

Septicemia is an important cause of treatment‐related mortality and treatment failure in pediatric acute lymphoblastic leukemia (ALL) in developing countries. A multicenter CCCG‐ALL‐2015 study was conducted in China and factors associated with septicemia and mortality were studied.

**Methods:**

Patients participated in CCCG‐ALL‐2015 study from January 2015 to December 2017 were included. Patients with documented septicemia were identified from the Data Center and additional data were collected.

**Results:**

A total of 4080 patients were recruited in the study and 527 patients with septicemia were identified (12.9%, 95% CI 11.9%‐13.9%). The intermediate risk (IR)/high risk (HR) group had significantly higher incidence of septicemia as compared with low risk (LR) group, 17.1% vs 9.1% (OR 2.07, 95% CI 1.71‐2.49, *P* < .001). Induction phase was the period with majority of septicemia episodes happened, 66.8% in LR and 56.1% in IR/HR groups. Gram‐positive bacteria accounted for 54.1%, gram‐negative bacteria 44.5%, and fungus 1.4% of positive cultures. Multidrug‐resistant organisms were detected in 20.5% of all organisms. The mortality rate after septicemia was 3.4% (95% CI 1.9%‐4.9%). Multiple logistic regression identified female gender, comorbid complications, and fungal infection as risk factors associated with mortality. Gram‐negative septicemia was associated with higher mortality, 4.9% vs 1.4% (OR 0.28, 95% CI 0.09‐0.88, *P* = .02). There was marked variation in the incidence of septicemia among the 18 centers, from 4.8% to 29.1%.

**Conclusion:**

Overall the incidence and pattern of septicemia in this multicenter study in China was similar to the reports of western countries. The septicemia‐related mortality rate was low. There was marked variation in the incidence of septicemia among the centers.

## Graphical abstract

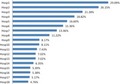

## 1 INTRODUCTION

Septicemia is one of the common complications after intensive chemotherapy, and it is also an important cause of treatment‐related mortality. With the widespread use of broad‐spectrum antibiotics, infection caused by multidrug‐resistant organisms is on the rising trend. The information of septicemia after chemotherapy in China is limited, and there is no national guidance on prevention and management of septicemia after chemotherapy. In January 2015, a multicenter study under China Children Oncology Group (CCCG) for acute lymphoblastic leukemia (ALL) was established, CCCG‐ALL‐2015 study. All the participating centers obtained ethical approval from their institutional ethics committee. The study had recruited 4080 subjects up to 31 December 2017. The study prospectively collected subjects' information including demographic data, leukemia‐related information, chemotherapy administration, treatment complications and treatment outcomes. There was also internal audit with at least once per year of site visit to study centers, and septicemia data were part of the mandatory monitoring during the audits. This study aimed at collecting data of documented septicemia, and analyzed the risk factors associated with the occurrence of septicemia and mortality. The result may provide guidance on preventive measures against septicemia in future studies.

## 2 METHODS

The chemotherapy protocol of CCCG‐ALL‐2015 study was based on St Jude Children's Research Hospital Total XV study with modification.^1^ Patients were stratified into low risk (LR), intermediate risk (IR), and high risk (HR) based on clinical, biological, and treatment responses. The study protocol did not include supportive care guidance on infection control or management. However, the protocol required mandatory co‐trimoxazole prophylaxis during chemotherapy except during high‐dose methotrexate phase. There were 20 centers participating in the study and patients' data were collected prospectively. Patients with documented septicemia were identified from the Study Data Center in early 2018 and further information about the septicemia were collected retrospectively from centers that agreed to participate in this sub‐study. Finally 18 centers could provide additional information for this septicemia study, including additional information on the clinical practice and infection control based on their center policies. Most centers followed international guidelines on management of infection and septicemia of cancer patients.^2^, ^3^, ^4^

### 2.1 Patients

From 1 January 2015 to 31 December 2017, eligible patients with diagnosis of ALL were recruited into CCCG‐ALL‐2015 study. Patients were below 18 years of age at the time of diagnosis. A written informed consent was obtained from patients or legal guardians. Other than patients' demographic data, leukemia information and compliance to chemotherapy, adverse events including septicemia were regular reporting events. The subjects with documented septicemia, including bacteria and fungus, were retrieved from the data file at the Study Data Center. Participation centers were invited to join this sub‐study and additional information not available in the core data file was collected.

### 2.2 Data collection

The Study Data Center retrieved the following data of documented septicemia: age, sex, risk stratification, chemotherapy phase at time of septicemia, comorbidities, and treatment outcome. Additional data were collected retrospectively for each subject from participation centers, including types of bacteria, sensitivity pattern, WBC and neutrophil counts at time of septicemia, concurrent morbidity, response to antimicrobials and outcome. Survey forms were also sent to participating centers to collect information about policy of infection management, including addition of extra beds in the wards, use of modular high efficiency particulate air (HEPA) filter isolation units, prophylactic use of antibiotics, intravenous immunoglobulin, and granulocyte colony‐stimulating factors (G‐CSF).

### 2.3 Chemotherapy treatment

Patients received multi‐agent chemotherapy at following treatment phases: induction (week 1‐7), consolidation (week 8‐15), continuation therapy and reinduction (week 16‐34), and maintenance phase (week 35‐125). Timing of septicemia at different phases of chemotherapy was analyzed. Concurrent morbidities and complications were also collected, namely pneumonia, peritonitis, respiratory failure, pancreatitis, liver derangement, gastrointestinal bleeding, septicemic shock, and cardiac failure.

Microbiological data based on blood cultures at the onset of fever were collected. Among the 18 centers, 11 centers performed blood cultures from both limbs, and most of the patients had peripheral inserted central catheters (PICC) during the intensive phases of chemotherapy, that is, one blood culture via PICC and the other from peripheral veins. These centers also inoculated blood samples in double bottles, aerobic and anaerobic. Seven centers collected blood culture from one site and the samples were inoculated into either single or double bottles. The volume of blood sample collected was according to instruction of suppliers and at least 3 mL of blood was collected. The detection for bacteria was by automatic detection systems. Eleven centers sent the specimen to laboratory within one hour, while seven centers sent to laboratory within 2 hours. The usual culture time was 5 to 7 days. Two centers had designated phlebotomists for blood collection, while nurses performing blood culture in the other centers.

### 2.4 Statistics

Data were analyzed using SPSS 16.0 statistical software. Categorical data were compared with chi‐squared test. Gender, Risk groups, and Ph ALL were tested for any differences for the incidence of septicemia. A univariate analysis was performed for risk factors leading to death, and multiple regression analysis would then be performed for those with significance. Fisher exact test was performed to comparison of different risk groups. *P* value < .05 would be considered as significant.

## 3 RESULTS

### 3.1 Demographic features

From 1 January 2015 to 31 December 2017, 4080 subjects were recruited into the CCCG‐ALL‐2015 study. There were 2438 males and 1642 females. Septicemia occurred in 527 patients, the incidence of septicemia was 12.9% (95% CI 11.9%‐13.9%) (527/4080). There was no difference in the incidence between genders, male 12.4% and female 13.7% (*P* > .05). The mean age at time of septicemia was 5.2 ± 3.8 years, and the median age was 4.0 years (range 0.3 to 16.7). Among the 527 patients, 30 patients developed septicemia twice during different phases of chemotherapy and one patient developed four episodes of septicemia at different phases with different organisms. Thus, a total of 560 episodes of septicemia were diagnosed.

### 3.2 Timing of septicemia

Among the 4080 patients recruited, 2150 patients were stratified as low risk (LR), and 1930 patients were diagnosed as intermediate or high risk (IR/HR). The incidence of septicemia was significantly higher among the IR/HR group as compared with LR group, 17.1% vs 9.1% (OR 2.06, 95% CI 1.71‐2.49, *P* < .001). Ph‐positive patients were stratified into IR/HR group and there were 166 Ph‐positive patients. Among the IR/HR group, the incidence of septicemia in Ph‐positive group was similar to Ph‐negative group, 20.5% vs 16.8% (OR 0.786, 95% CI 0.528‐1.169, *P* = .234). Of 205 septicemia episodes among the LR group, a majority of septicemia episodes occurred during the induction and consolidation phase. For the 355 episodes of septicemia in IR/HR group, induction phase was also the period with highest percentage of septicemia, reinduction phase was another peak period with septicemia. The reinduction phase in IR/HR group included high‐dose cytarabine. The timing of septicemia during different treatment phases is shown in Table 1.

**Table 1:** Timing of septicemia in relation to treatment phase

	Low risk (n = 2150)	Intermediate/high risk (n = 1930)
No. of patients with septicemia	196 (9.1%)	331 (17.1%)
No. of septicemia episodes	205	355
Induction (week 1‐7)	137 (66.8%)	199 (56.1%)
Gram positive	82	118
Gram negative	60	94
Consolidation (week 8‐15)	29 (14.1%)	26 (7.3%)
Gram positive	16	15
Gram negative	13	11
Continuation and reinduction (week 16‐34)	19 (9.3%)	90 (25.4%)
Gram positive	14	48
Gram negative	5	45
Maintenance (week 35‐125)	20 (9.8%)	40 (11.3%)
Gram positive	9	18
Gram negative	12	23

### 3.3 Organisms isolated from blood culture

Gram‐positive bacteria was more commonly isolated, 54.1% (320 isolates), and gram‐negative organisms accounted for 44.5% (263 isolates). There were eight fungal isolates, four were yeast like, three candida species, one *Penicillium marneffei*. There were 20.5% (121 isolates) being multidrug resistant strains. The top 10 isolates were coagulase‐negative staphylococci (20.1%), *Staphylococcus epidermidis* (14.6%), *E coli* (11.5%, 29/68 were ESBL), *klebsiella pneumoniae* (8%, 7/47 were ESBL), *Pseudomonas aeruginosa* (7%, 2/41 were ESBL), *Staphylococcus aureus* (5.6%, 8/33 were MRSA), *Streptococcus mitis* (3.2%), *Streptococcus pneumoniae* (3%), salmonella (2%), and *Enterobacter cloacae* (1.7%). Multidrug‐resistant organisms were detected in 20.5% of all organisms.

### 3.4 Mortality and risk factors

Among 560 episodes of septicemia, there were 19 cases of death and the mortality rate was 3.4%. Among the 19 deaths, 12 were of IR/HR group and 7 were LR group. The timing of septicemia related death were at induction/consolidation phase in 12 cases, and another 7 cases during continuation phase. When the analysis was performed separately for LR and IR/HR groups at different treatment phases, there was no significant difference of mortality rate during the different chemotherapy phases for both of the two groups. Organisms isolated in patients with septicemia‐related death were *E coli* (n = 7), coagulase‐negative staphylococcus (n = 5), *Pseudomonas aeruginosa* (n = 3), *Candida tropicalis* (n = 2). The other organisms were haemophilus, *Klebsiella pneumoniae*, *Streptococcus mitis*, and *Staphylococcus aureus*. Gram‐negative bacteria accounted for mortality in 57.1% and gram‐positive 33.3% and fungus 9.5%.

Univariate analysis identified the following risk factors associated with death: female gender (5.4% vs 1.9%, *P* = .02), severe neutropenia with neutrophils <0.1 × 10^9^/L (4.6% vs 1.1%, *P* = .02), presence of concurrent comorbidities (16.3% vs 1.1%, *P* < .001), gram‐negative bacteria (4.9% vs 1.4%, *P* = .02). The other factors were not found to be significantly associated with mortality: risk group, neutropenia more than 7 days, presence of fever, multiple organisms isolated in the blood culture (Table 2). The logistic regression analysis of the above factors was performed, and female gender, associated comorbidities and fungal infection were the significant factors (Table 3). Among the 560 episodes of septicemia, 37 episodes have multiple organisms isolated or fungal isolated. For the 527 episodes with single isolates of gram‐negative or gram‐positive organisms, gram‐negative septicemia was associated with higher death rate, 4.9% vs 1.9%. (OR0.281, 95% CI 0.089‐0.884, *P* = .02). Among the 560 episodes of septicemia, concurrent comorbidities were noted in 86 patients. Septicemia shock was present in 20 cases, pancreatitis 14 cases, liver derangement 11 cases, electrolyte disturbances in 9 cases, disseminated intravascular coagulation in 7 cases.

**Table 2:** Mortality rate and risk factors

Group	N = 560	Death (n = 19)	*P* value	OR	95% CI for OR	
					Lower	Upper
Gender						
Male	318	6 (1.9%)	.02	2.95	1.11	7.88
Female	242	13 (5.4%)				
Risk group						
LR	205	7 (3.4%)	.98	0.99	0.38	2.56
IR/HR	355	12 (3.4%)				
Fever						
Yes	556	19 (3.4%)	1.00	0.97	0.95	0.98
No	4	0 (0%)				
Severe neutropenia^a^						
Yes	369	17 (4.6%)	.03	0.22	0.05	0.96
No	191	2 (1.1%)				
Neutropenia ≥7 d						
Yes	187	6 (3.2%)	.87	1.09	0.41	2.92
No	373	13 (3.5%)				
Multidrug resistant						
Yes	121	7 (5.8%)	.15	0.46	0.18	1.19
No	439	12 (2.7%)				
Concurrent comorbidities						
Yes	86	14 (16.3%)	<.001	0.06	0.02	0.16
No	474	5 (1.1%)				
Fungal infection						
Yes	8	2 (25%)	.03	0.10	0.02	0.51
No	552	17 (3.1%)				
Multiple organisms						
Yes	31	2 (6.5%)	.28	0.48	0.11	2.18
No	529	17 (3.2%)				
Gram stain^b^						
Positive	279	4 (4.9%)	.02	0.28	0.09	0.88
Negative	244	12 (1.4%)				

a Neutrophil counts <0.1 × 10^9^/L.

b Only limited to pure growth cases.

**Table 3:** Multiple logistic regression on factors related to death after septicemia

	B	SE	Wald	df	sig	Exp(B)	95% CI for Exp(B)	
							Lower	Upper
Gender	−1.23	0.54	5.17	1	0.02	0.29	0.10	0.84
Comorbidities	2.96	0.59	24.31	1	<0.001	19.22	5.94	62.22
Fungemia	3.57	1.18	9.18	1	0.002	35.57	3.53	358.37
Severe neutropenia	0.77	0.79	0.93	1	0.34	2.15	0.45	10.25
Gram negative	1.03	0.62	2.76	1	0.09	2.81	0.83	9.52

### 3.5 Center variability in septicemia incidences

**Figure 1:**
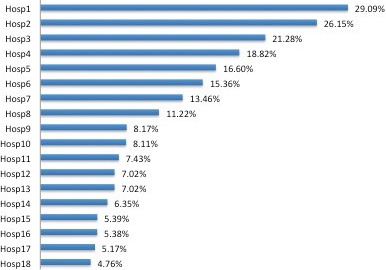
Incidences of septicemia among the 18 centers

Figure 1 shows the variation in the incidences of septicemia among the 18 centers, from 4.8% to 29.1%. Subsequent survey to the participating centers obtained further information for analysis. Center policies appeared to affect the incidence of septicemia. Centers allowed additional beds opened in the ward due to full occupancy had higher incidence of septicemia (Table 4). Centers with practice of using modular HEPA filter units in the wards for patients with severe neutropenia were having lower incidence of septicemia. Prophylactic use of G‐CSF at time of neutropenia (neutrophils <0.5 × 10^9^/L) was also associated with lower incidence of septicemia. Similarly, prophylactic antibiotics and intravenous immunoglobulin were also associated with lower incidence of septicemia. When analysis was done on association of above factors with death, using modular HEPA filter was found to be the only factor significantly associated with death.

**Table 4:** Center policies and septicemia

Factors	Septicemia (n = 527)	*P* value	OR	95% CI for OR		Death (n = 19)	*P* value	OR	95% CI for OR	
				Lower	Upper				Lower	Upper
Additional beds										
Yes	326 (16.7%)	<.001	0.52	0.43	0.63	15 (4.6%)	.12	0.42	0.14	1.29
No	201 (9.5%)					4 (2.0%)				
HEPA units										
Yes	95 (6.7%)	<.001	2.69	2.13	3.39	9 (9.5%)	.003	0.23	0.09	0.57
No	432 (16.2%)					10 (2.3%)				
GCSF										
Prophylactic	153 (9.4%)	<.001	1.73	1.42	2.11	6 (3.9%)	.80	0.88	0.33	2.37
No prophylactic	374 (15.2%)					13 (3.5%)				
IVIG										
Prophylactic	76 (8.7%)	<.001	1.73	1.34	2.23	4 (5.3%)	.40	0.62	0.20	1.92
No prophylactic	451 (14.1%)					15 (3.3%)				
Prophylactic antibiotics										
Yes	68 (7.0%)	<.001	2.30	1.76	2.99	5 (3.3%)	.08	0.39	0.14	1.14
No	459 (14.8%)					14 (3.7%)				

## 4 DISCUSSION

ALL is the commonest childhood malignancy and the 5‐year event‐free survival (EFS) is now approaching 90% in developed countries.^5^, ^6^, ^7^ However, the survival outcome is still suboptimal in developing countries.^8^, ^9^ A recent multicenter study from China reported 5‐year EFS of 79.9%.^10^ Septicemia is an important cause of treatment‐related mortality. St Jude Children's Research Hospital reported 78% 10‐year survival with the Total XIII Study, and second malignancy was the most important cause of treatment failure.^11^ In Turkey, a similar Total XIII protocol was adopted and the 5‐year EFS was 77%. The induction death was 12.3% and infection was the most important cause of death.^12^ In India, septicemia was also reported to be an important cause of death, accounting for 29.7% of deaths.^13^ In the multicenter report of ALL from China, non‐relapse mortality during remission was 2.9% and infection was the cause of death in 73.4%.^10^ Severe infection during chemotherapy is associated with mortality and also increase in medical cost; from prolonged hospitalization, ICU care, use of expensive antibiotics, and other supportive treatment. The high treatment cost is one of the prohibitory factor for completion of chemotherapy treatment in developing countries. In USA, a study showed that patients with ALL and septicemia had more than double of the treatment expense due to longer hospitalization.^14^ Septicemia increases the medical expense and economic burden of the families, and prevention of septicemia may facilitate the completion of planned treatment and thus the cure rate.

The overall incidence of septicemia in this study was 12.9%. A study of 6220 patients from USA reported the incidence of septicemia of 12.6%, however, the study included also patients with acute myeloid leukemia.^14^ In the previous reports of ALL studies in China, septicemia was reported at higher incidence and also an important cause of death.^15^ In this study, patients treated with IR/HR arm chemotherapy was associated with higher incidence of septicemia (17.1% vs 9.1%, *P* < .001). The IR/HR group received more intensive treatment, the more severe marrow suppression led to higher chance of infection. Ph‐positive patients received IR arm chemotherapy combined with tyrosine kinase inhibitors, imatinib or dasatinib. There was previous report of higher incidence of infection in Ph ALL patients treated with combination of tyrosine kinase inhibitor and chemotherapy. EsPhALL 2010 study reported 31% of patients developed infection.^16^ In the current study, we did not observe higher incidence of septicemia in Ph ALL patients as compared with other non‐Ph positive IR patients, 20.5% vs 16.8% (*P* = .23). The higher incidence of infection in Ph‐positive cases in other study might be related to a more intensive chemotherapy backbone.

At the induction phase, patients were at highest risk of infection due to the underlying active leukemia and intensive chemotherapy. During the induction phase, patients also underwent many invasive procedures such as PICC insertion, bone marrow aspiration, and lumbar puncture. The family might not be familiar with the infection control measures enhancing education to parents on infection control and sterile procedures are necessary. The other peak period of septicemia in IR/HR group was at re‐induction phase at week 32‐34. This phase of treatment included longer period of high‐dose dexamethasone and high‐dose cytarabine which may lead to more severe mucosal damage and secondary infection. Prophylactic antibiotics and close monitoring in hospital may lower the high rate of septicemia. For patients already discharged from hospitals, education on early warning signs of infection and seeking early medical attention may reduce mortality. Doctors at Emergency Departments should receive training on prompt management of patients with febrile neutropenia.

The types of isolates reported in literature varied with centers. Centers exposed more frequently to antibiotics are associated with more gram‐positive organisms, and also more commonly with resistant strains of gram‐negative organisms.^17^, ^18^ In the current study, there was higher percentage of gram‐positive organism isolated. The types of organisms isolated were similar to other reports, coagulase‐negative staphylococcus and *Staphylococcus epidermidis* were the top two organisms, followed by *E coli* and klebsiella. Fungemia was identified in only eight cases and this might be underestimated as isolation of fungus in blood culture usually has lower yield. The choice of antibiotics usually based on the clinical conditions and past experience of drug sensitivity pattern in the centers. The 2010 IDSA guideline recommended the monotherapy of lactamase antibiotics, piperacillin, and carbapenam. However, for patients with chest infection or soft tissue infection, vancomycin should be instituted early.^2^ In this study, 20.5% of the isolates were multidrug resistant. *E coli* isolates had a high ESBL positivity at 42.7% and the klebsiella also had 14.9% being ESBL positive. MRSA was also prevalent in 24.3% of *Staphylococcus aureus* isolates. Early switch or addition of carbepenam or vancomycin should be considered in centers with high incidence of resistant strains.

The death rate from septicemia in the current study was 3.2%. In a previous report from Shanghai 10 years ago, the death rate was 15.5%.^19^ The improvement in outcome of septicemia is probably related to early recognition of sepsis and institution of potent antibiotics. Logistic regression analysis for the factors associated with death was performed. Female gender, fungal infection, and the presence of comorbidities were the risk factors to death. The reason why females had higher death rate (5.4% vs 1.9%) was not clear. The presence of comorbidities with impaired organ function put the patients at higher risk of death. In the 2010 IDSA guideline, the presence of comorbidities such as hypotension, pneumonia, and neurological symptoms are also regarded as high risk with poor prognosis.^2^ The positive fungal culture was low in the study, but it was associated with 25% mortality. Use of prophylactic anti‐fungal agents may be considered but there is chance of increase in drug resistant. Gram‐negative septicemia was associated with higher death rate, 4.9% vs 1.4%. Gram‐negative septicemia are more aggressive and there are also more resistant strains, early admission into ICU for close monitoring should be considered.

In the CCCG‐ALL‐2015 study, there was marked variability of incidence of septicemia, from 4.8% to 29.1%. In recent years in China, some centers could not cope with the high service demand and the hematology wards were overcrowded. Addition of extra beds in the wards have been practiced in some centers. Some centers also equipped with modular HEPA filter units in the wards trying to reduce infection. Prophylactic use of quinolones to prevent infection after chemotherapy have been reported to be successful.^20^, ^21^, ^22^ However, quinolone is not allowed to be used in children in China yet. Some centers started to use beta lactamase antibiotics as prophylaxis during the neutropenia phase. Prophylactic use of G‐CSF after achieving remission and intravenous immunoglobulin was also practiced in some centers. The above measures appeared to have beneficial effects of reducing incidence of septicemia in our cohort. However, these measures should be studied in a more scientific approach, and randomized control trials should be performed.

Our study is the largest childhood ALL study in China with over 4000 patients recruited. We collected the diagnosis of septicemia prospectively with regular internal audit to confirm the validity. However, the information on septicemia from this research study might not reflect the real world situation in non‐research setting. The septicemia‐related information was collected retrospectively which might not be complete and with recall bias. The wide variation in the incidence of septicemia among the centers suggested center effect.

In summary, the incidence and pattern of septicemia in this multicenter study in China was similar to reports of western countries. The septicemia‐related mortality rate was low. There was marked variation in the incidence of septicemia among the centers which might be related to different practice of infection prophylaxis. Further studies are required to look into factors which might influence the septicemia incidence and mortality.

## Data Availability

The data that support the findings of this study are available from the corresponding author upon reasonable request.

## References

[1] Cai J , Yu J , Zhu X , et al.; Chinese Children's Cancer Group childhood acute lymphoblastic leukaemia (ALL) 2015 study group (CCCG‐ALL‐2015) . Treatment abandonment in childhood acute lymphoblastic leukaemia in China: a retrospective cohort study of the Chinese Children's Cancer Group. Arch Dis Child. 2019;104(6):522‐529. 30705079 10.1136/archdischild-2018-316181

[2] Freifeld AG , Bow EJ , Sepkowitz KA , et al. Clinical practice guideline for the use of antimicrobial agents in neutropenic patients with cancer: 2010 update by the Infectious Disease Society of America. Clin Infect Dis. 2011;15(52):e56‐e93. 10.1093/cid/cir073 21258094

[3] Rolston KV . Neutropenic fever and sepsis: evaluation and management. Cancer Treat Res. 2014;161:181‐202. 24706225 10.1007/978-3-319-04220-6_6

[4] Lehrnbecher T , Robinson P , Fisher B , et al. Guideline for the management of fever and neutropenia in children with cancer and hematopoietic stem‐cell transplantation recipients: 2017 update. J Clin Oncol. 2017;35:2082‐2094. 28459614 10.1200/JCO.2016.71.7017

[5] Pui C‐H , Yang JJ , Hunger SP , et al. Childhood acute lymphoblastic leukemia: progress through collaboration. J Clin Oncol. 2015;33:2938‐2948. 26304874 10.1200/JCO.2014.59.1636 PMC4567699

[6] Bonaventure A , Harewood R , Stiller CA , et al. Worldwide comparison of survival from childhood leukaemia for 1995–2009, by subtype, age, and sex (CONCORD‐2): a population‐based study of individual data for 89 828 children from 198 registries in 53 countries. Lancet Haematol. 2017;4(5):e201. 10.1016/S2352-3026(17)30052-2 PMC5418564 28411119

[7] Tai EW , Ward KC , Bonaventure A , Siegel DA , Coleman MP . Survival among children diagnosed with acute lymphoblastic leukemia in the United States, by race and age, 2001 to 2009: findings from the CONCORD‐2 study. Cancer. 2017;123(Suppl 24):5178‐5189. 29205314 10.1002/cncr.30899 PMC6075705

[8] Rivera GK , Ribeiro RC . Improving treatment of children with acute lymphoblastic leukemia in developing countries through technology sharing, collaboration and partnerships. Expert Rev Hematol. 2014;7:649‐657. 25174644 10.1586/17474086.2014.949233 PMC4174393

[9] Abdelmabood S , Fouda AE , Boujettif F , Mansour A . Treatment outcomes of children with acute lymphoblastic leukemia in a middle‐income developing country: high mortalities, early relapses, and poor survival. J Pediatr. 2018 10.1016/j.jped.2018.07.013 PMC9432263 30240631

[10] Cui L , Li Z‐G , Chai Y‐H , et al. Outcome of children with newly diagnosed acute lymphoblastic leukemia treated with CCLG‐ALL 2008: the first nation‐wide prospective multicenter study in China. Am J Hematol. 2018;93:913‐920. 29675840 10.1002/ajh.25124

[11] Pui C‐H , Boyett JM , Rivera GK , et al. Long‐term results of total therapy studies 11, 12 and 13A for childhood acute lymphoblastic leukemia at St Jude Children's Research Hospital. Leukemia. 2000;14:2286‐2294. 11187920 10.1038/sj.leu.2401938

[12] Koc A , Aycicek A , Ozdemir ZC , Soker M , Varma M . Outcome of modified St Jude total therapy 13A for childhood acute lymphoblastic leukemia in the southeast region of Turkey. J Pediatr Hematol Oncol. 2013;35:36‐41. 23138117 10.1097/MPH.0b013e318271f43f

[13] Marwaha RK , Kulkarni KP , Bansal D , Trehan A . Pattern of mortality in childhood acute lymphoblastic leukemia: experience from a single center in northern India. J Pediatr Hematol Oncol. 2010;32:366‐369. 20502353 10.1097/MPH.0b013e3181e0d036

[14] Allareddy V , Rampa S , Allareddy V . Hospital charges and length of stay associated with septicemia among children hospitalized for leukemia treatment in the United States. World J Pediatr. 2012;8:222‐228. 22886194 10.1007/s12519-012-0361-5

[15] Gu LJ , Li J , Xue HL , et al. Clinical outcome of children with newly diagnosed acute lymphoblastic leukemia treated in a single center in Shanghai, China. Leuk Lymphoma. 2008;49:488‐494. 18297525 10.1080/10428190701784730

[16] Biondi A , Gandemer V , De Lorenzo P , et al. Imatinib treatment of paediatric Philadelphia chromosome‐positive acute lymphoblastic leukaemia (EsPhALL2010): a prospective, intergroup, open‐label, single‐arm clinical trial. Lancet Haematol. 2018;5:e641‐e652. 30501871 10.1016/S2352-3026(18)30173-X

[17] Ramphal R . Changes in the etiology of bacteremia in febrile neutropenic patients and the susceptibilities of the currently isolated pathogens. Clin Infect Dis. 2004;39(Suppl 1):S25‐S31. 15250017 10.1086/383048

[18] Cattaneo C , Quaresmini G , Casari S , et al. Recent changes in bacterial epidemiology and the emergence of fluoroquinolone‐resistant *Escherichia coli* among patients with haematological malignancies: results of a prospective study on 823 patients at a single institution. J Antimicrob Chemother. 2008;61:721‐728. 18218645 10.1093/jac/dkm514

[19] Gao YJ , Lu FJ , Wang HS . Treating childhood acute lymphoblastic leukemia in a developing country 1998–2003: the experience of a single children's hospital in China. J Pediatr Hematol Oncol. 2006;28:789‐802. 10.1097/MPH.0b013e31802d3e7a 17164648

[20] Sulis ML , Blonquist TM , Stevenson KE , et al. Effectiveness of antibacterial prophylaxis during induction chemotherapy in children with acute lymphoblastic leukemia. Pediatr Blood Cancer. 2018;65:e26952. 29319209 10.1002/pbc.26952

[21] Wolf J , Tang LI , Flynn PM , et al. Levofloxacin prophylaxis during induction therapy for pediatric acute lymphoblastic leukemia. Clin Infect Dis. 2017;65:1790‐1798. 29020310 10.1093/cid/cix644 PMC5850441

[22] Alexander S , Fisher BT , Gaur AH , et al. Effect of levofloxacin prophylaxis on bacteremia in children with acute leukemia or undergoing hematopoietic stem cell transplantation: a randomized clinical trial. JAMA. 2018;320:995‐1004. 30208456 10.1001/jama.2018.12512 PMC6143098

